# Chondroprotection by urocortin involves blockade of the mechanosensitive ion channel Piezo1

**DOI:** 10.1038/s41598-017-04367-4

**Published:** 2017-07-11

**Authors:** K. M. Lawrence, R. C. Jones, T. R. Jackson, R. L. Baylie, B. Abbott, B. Bruhn-Olszewska, T. N. Board, I. C. Locke, S. M. Richardson, P. A. Townsend

**Affiliations:** 1 Division of Cancer Sciences, Manchester Cancer Research Centre, Manchester Academic Health Sciences Centre, The University of Manchester, Wilmslow Road, Manchester, M20 4GJ UK; 2 Division of Cardiovascular Sciences, Manchester Academic Health Sciences Centre, University of Manchester, M13 9NT Manchester, UK; 3 The Center for Hip Surgery, Wrightington Hospital, Wigan, WN6 9EP UK; 4 Department of Biomedical Sciences, University of Westminster, London, W1W 6UW UK; 5 Division of Cell Matrix Biology and Regenerative Medicine, Centre for Tissue Injury and Repair, Manchester Academic Health Sciences Centre, University of Manchester, Manchester, M13 9PT UK

## Abstract

Osteoarthritis (OA) is characterised by progressive destruction of articular cartilage and chondrocyte cell death. Here, we show the expression of the endogenous peptide urocortin1 (Ucn1) and two receptor subtypes, CRF-R1 and CRF-R2, in primary human articular chondrocytes (AC) and demonstrate its role as an autocrine/paracrine pro-survival factor. This effect could only be removed using the CRF-R1 selective antagonist CP-154526, suggesting Ucn1 acts through CRF-R1 when promoting chondrocyte survival. This cell death was characterised by an increase in p53 expression, and cleavage of caspase 9 and 3. Antagonism of CRF-R1 with CP-154526 caused an accumulation of intracellular calcium (Ca^2+^) over time and cell death. These effects could be prevented with the non-selective cation channel blocker Gadolinium (Gd^3+^). Therefore, opening of a non-selective cation channel causes cell death and Ucn1 maintains this channel in a closed conformation. This channel was identified to be the mechanosensitive channel Piezo1. We go on to determine that this channel inhibition by Ucn1 is mediated initially by an increase in cyclic adenosine monophosphate (cAMP) and a subsequent inactivation of phospholipase A_2_ (PLA_2_), whose metabolites are known to modulate ion channels. Knowledge of these novel pathways may present opportunities for interventions that could abrogate the progression of OA.

## Introduction

Articular cartilage is the dense connective tissue that lines the surfaces of diarthrodial joints providing a low-friction surface for joint loading and articulation. The extracellular matrix of articular cartilage comprises primarily of proteoglycans and type II collagen, which are maintained by a sparse population of chondrocytes^1^. Osteoarthritis (OA) is characterised by progressive destruction and loss of cartilage, which is attributed to a reduction in the number of viable chondrocytes in articular cartilage^2^ and the severity of cartilage damage has been shown to correlate negatively with the number of remaining chondrocytes^3^. Chondrocyte cell death is essentially apoptotic in nature^4^, with a close correlation between p53 expression and death^5^. Currently, the only treatments for OA are steroidal and non-steroidal anti-inflammatory drugs, or in severe cases total joint replacement surgery^6^. However, these strategies only ameliorate symptoms and do not address the underlying pathology, namely chondrocyte death. The prevention of this death and/or the protection of remaining cells from further destruction would represent a treatment strategy that addresses important cartilage degrading diseases such as OA at a more fundamental level. Despite the fact that chondrocyte cell death is now well established as a contributing factor in the loss of articular cartilage integrity, the cause of this death is currently unclear. However, several molecular factors have been implicated, including nitric oxide (NO), which has been shown to induce death *in vitro* and *in vivo* ^7, 8^. Furthermore, NO is abundant in OA joints *in vivo* and is thought to be elevated by mechanical stress^9^.

We have recently demonstrated the expression in the human chondrocyte cell line C-20/A4 of the corticotropin-releasing factor (CRF)-related peptide urocortin 1 (Ucn1). Furthermore, we demonstrated that the addition of exogenously applied Ucn1 to C-20/A4 cells was able to protect against NO-induced apoptosis. Intriguingly, we also discovered that Ucn1 works as an essential endogenous autocrine pro-survival molecule in the absence of apoptotic stimuli, since its removal from the surrounding milieu in cultured cells caused cell death^10^. Significantly, Ucn1 has recently been found to be upregulated in the synovial fluid of patients with rheumatoid arthritis and has been shown to reduce inflammation in mouse models of the disease^11–13^.

Ucn1 is a 40 amino acid long peptide and was cloned based on sequence homology to CRF, the parent molecule^14^. These peptides are evolutionary ancient molecules having representatives in lower vertebrates such as sauvagine and urotensin, found in amphibia and fish respectively^15, 16^. Although originally found in the brain, Ucn1 has now been found in many peripheral tissues where it exerts diverse effects including: cardioprotection^17^, antiresorptive activity in bone^18^ and control of the myometrium at term^19^. Two further paralogues of Ucn1 have also been isolated; Ucn2 (Human Stresscopin Related Peptide), and Ucn3 (Human Stresscopin), which are composed of 38 and 39 amino acids respectively^20^. All ligands transduce signals by binding to two different G protein-linked receptor subtypes CRF R1 and CRF-R2. Furthermore, ligand binding studies have revealed that CRF and Ucn1 have affinity for both receptor subtypes, whereas Ucn2 and Ucn3 bind exclusively to CRF-R2^21^. This system is completed by corticortropin releasing factor-binding protein (CRF-BP), which acts as a decoy receptor for both CRF and Ucn1^22, 23^, implying that this family of receptors and ligands may be self-regulating.

There is a growing body of evidence implicating Ucn1 in ion channel modulation in different cell types and under different physiological conditions^24^. Ucn1 can affect a whole range of ion channel species in different tissues resulting in profound physiological effects. For example in the vasculature, Ucn1 has been demonstrated to relax the endometrial smooth muscle by activation of large Ca^2+^-activated K^+^ channels (BK channels)^25^. Whereas the cardioprotective effect of Ucn1 involves a diverse range of ion channels including the inhibition of L-type calcium channels^26^ but also to increase KATP channel gene expression and activation^17, 27^. Furthermore, in the male reproductive system, the reported regulation of spermatogenesis by Ucn1 has been demonstrated to involve T-type Ca^2+^ channels^28^, whereas the osteoclast inhibitory effect of Ucn1 involves regulation of Ca^2+^ ions by inhibition of a TRPC1-like cation current^18^.

The presence of ion channels on chondrocytes, the so called chondrocyte “channelome” is of growing interest within the field of OA^29^. A number of ion channels have been shown to be expressed on the surface of the chondrocytes, which contribute to the metabolic activity of the cell. This activity is essential for induction and production of the ECM proteins in response to mechanical damage and stress^30^. The importance of calcium influx through these channels has also been described, in particular in response to mechanical impact. Gene knockout of a T-type calcium channel expressed on both osteoblasts and chondrocytes in a mouse model was shown to reduce OA histology scores^31^. Furthermore, membrane ion channel characteristics and expression have been shown to change with development of OA^32, 33^ and specifically the acid sensing potassium channel (TASK-2), epithelial sodium channel (ENaC) and Ca^2+^ activated chloride channel (anoctamin-1, TMEM16) have all been shown to be decreased in OA patients compared to healthy individuals. Conversely Ca^2+^ activated potassium channels (KCa3.1, “SK” and KCa1.1, “BK”) and aquaporin 1 (AQP1) were shown to be strongly up-regulated^32, 33^. Recently, the newly discovered, Piezo1 and Piezo2 ion channels, have been found in cartilage and are thought to represent important chondrocyte mechanotransduction pathways. These ion channels are large mechanically activated Ca^2+^-permeable cation channels and are both abundant in organs with mechanical functions including chondrocytes^34–38^.

The aim of our study was to elucidate the pathway by which Ucn1 protects chondrocytes. To this end we used the C-20/A4 cell line to first identify the biological mechanisms of Ucn1 protection before confirming key experiments in primary human articular chondrocytes (AC). The C-20/A4 phenotype and gene expression profiling in both monolayer and 3D alginate culture systems, confirmed their suitability as an appropriate cell line to use in the study of chondrocyte biology^39^. Furthermore, C-20/A4 cells display the polygonal cobblestone morphology and the differentiated phenotype typical of human chondrocytes such as the expression of various cartilage specific molecules including collagen type II, IX and XI^40^.

We report here that the Ucn1 system (Ucn1, CRF-R1 and CRF-R2) is expressed in AC and to confirm that Ucn1 is essential for the survival of AC cells in the absence of pro-apoptotic stimuli. Furthermore, we have elucidated the downstream signalling pathway by which Ucn1 exerts this protective effect and the end effector ion channel subtype as Piezo1. These findings provide extremely important and novel insights into the development of treatments for OA. These treatments rather than being simply palliative will address the fundamental problem of maintaining chondrocyte survival. Therefore, pharmaceuticals derived from this work could be potentially prophylactic as well as halting the progression of the extant disease.

## Results

### The Urocortin system is present in primary human AC

**Figure 1:**
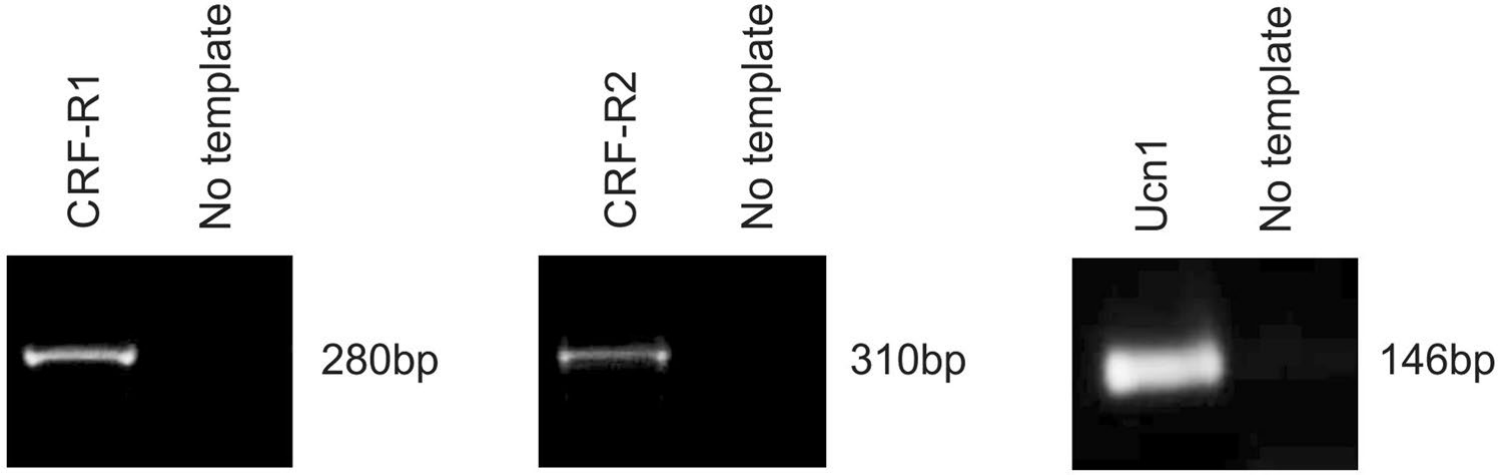
Analysis of the Ucn1 system in AC. RT-PCR analysis of transcripts derived from AC revealed the presence of Ucn1, CRF-R1 and CRF-R2 at the mRNA level. Predicted amplicon sizes and PCR conditions are summarised in Supplementary Table S1.

Having previously established that components of the Ucn1 signalling axis (Ucn1, CRF-R1 and CRF-R2) are expressed in the human chondrocyte cell line C-20/A4^10^ we sought here to examine the presence and validate this system in AC using RT-PCR. Analysis confirmed that Ucn1 itself and both the CRF-R1 and CRF-R2 receptors were expressed in AC (Fig. 1).

### The pro-survival effect of Ucn1 in both C-20/A4 and AC is mediated through the CRF-1 receptor

We have previously demonstrated that Ucn1 protects the C-20/A4 cell line from NO-induced cell death and is essential for cell survival in the absence of exogenously applied pro-apoptotic stimuli^10^.

**Figure 2:**
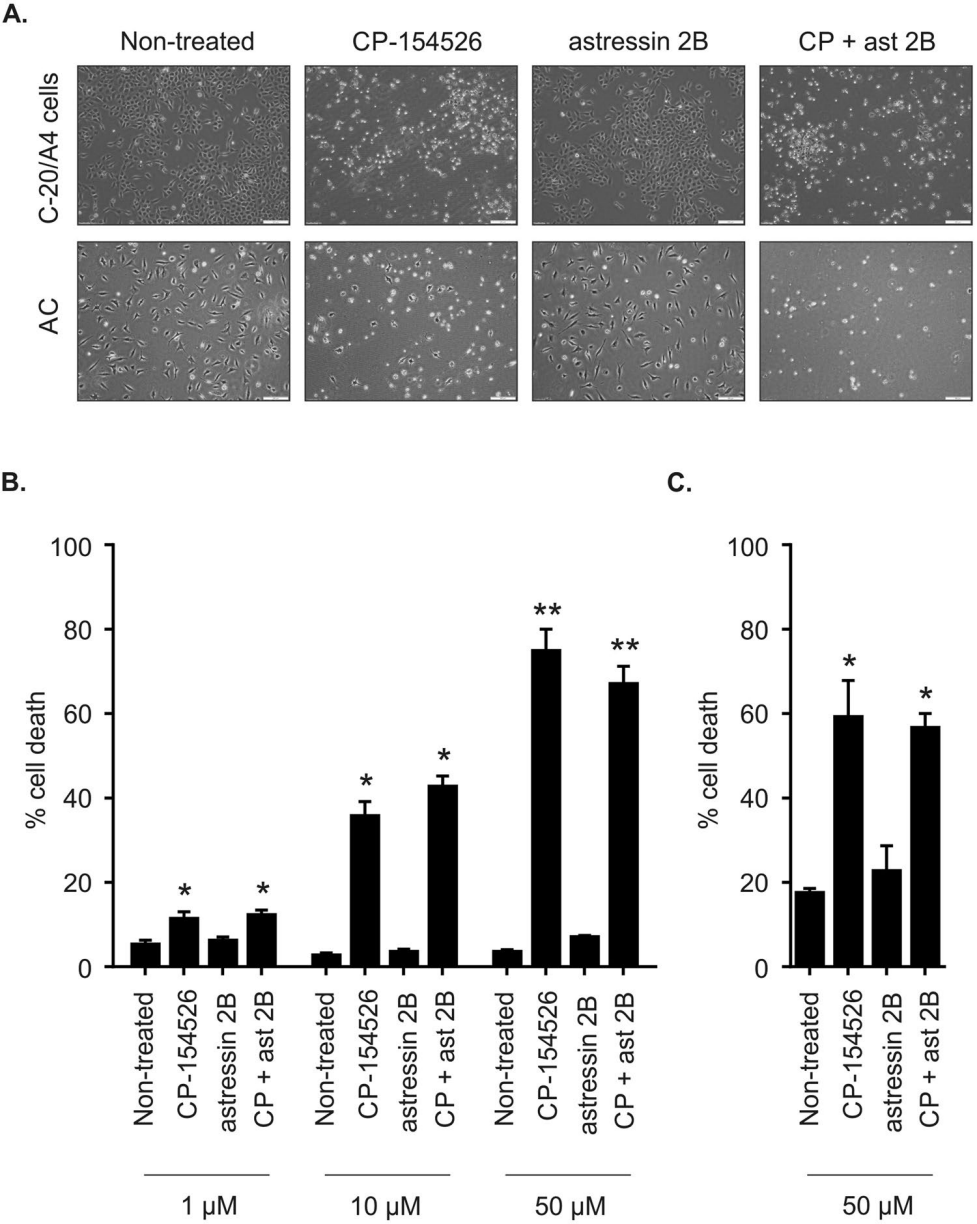
Response of C-20/A4 cells and AC to Ucn1 blockade. Cells were treated for 8 h with CP-154526, astressin 2B or in combination at indicated concentrations. (**A**) The effects on morphology of incubating C-20/A4 cells and AC with CP-154526 and/or astressin 2B (1–50 μM) was assessed using phase contrast microscopy. (**B**) Dose dependant effects of CP-154526 and/or astressin 2B on C-20/A4 cells. (**C**) AC cell death as determined by Annexin V/PI staining and flow cytometry. n = 3, error bars = SD *p < 0.05 and **p < 0.005 compared with untreated control cells.

Using specific antagonists against CRF-R1 (CP-154526) and CRF-R2 (astressin 2B) receptors we demonstrate a dose dependant increase in cell death after 8 h. C-20/A4 cells exhibited a rounded rather than a flattened morphology and became increasingly dislodged from the culture dish. Cell death was confirmed using Annexin V/PI staining with flow cytometry (Fig. 2A,B). A similar level of cell death was observed when cells were exposed to both CP-154526 and astressin 2B in combination, suggesting that the effect is not cumulative and that CRF-R1 is the sole receptor responsible for the demonstrated effects. Having established the CRF-R1 receptor as being responsible for Ucn1 mediated protection in C-20/A4 cells, we investigated the effect of Ucn1 blockade on AC and found Ucn1 also acts as a pro-survival molecule in AC through CRF-R1 (Fig. 2A,C).

### CRF-R1 blockade upregulates p53 and activates the intrinsic apoptotic pathway

**Figure 3:**
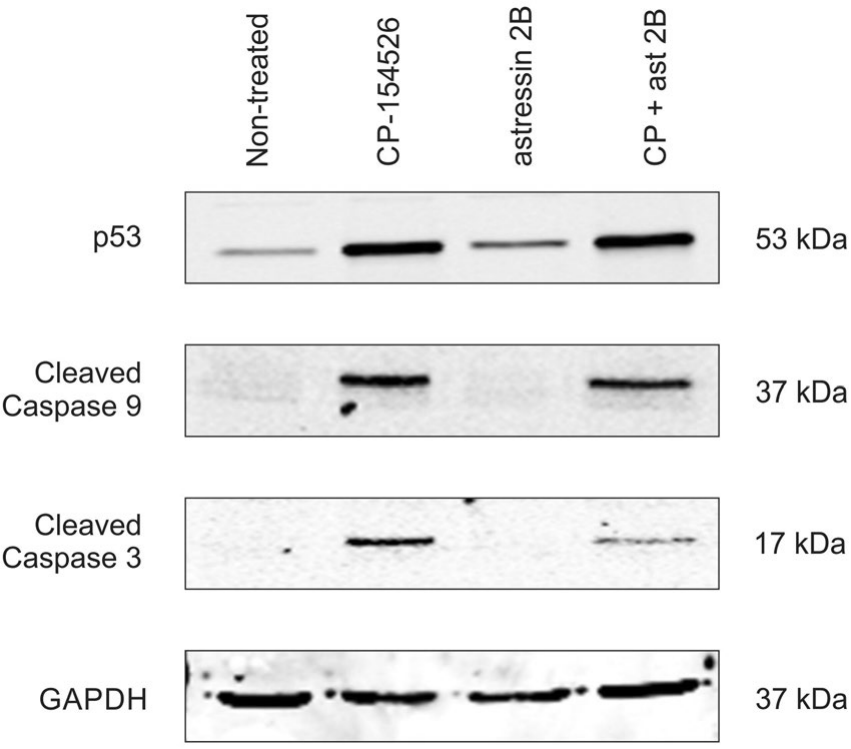
Western immunoblot analysis of C-20/A4 cells treated with CP-154526 and/or astressin 2B. Protein extracts were electrophoresed and probed with the indicated primary antibodies. Expected molecular sizes are indicated. p53 was upregulated in response to CP-154526 (50 μM) but not astressin 2B (50 μM). Cleaved caspases 3 and 9 were detected in the presence of CP-154526 but not in the presence of astressin 2B or non-treated cells. The images have been cropped but gels were run under the same experimental conditions. Representative images of 3 independent experiments.

To determine the mechanism of cell death caused by the addition of the CRF-R1 antagonist, we assessed the levels of p53, and cleaved-caspases 3, 8 and 9 in C-20/A4 cells by western blotting. When the cells were treated with CP-154526, p53 expression was up regulated and cleavage products for both caspases 9 and 3 were detected; cleaved-caspase 8, however, was not detected (data not shown). No effects were seen with the CRF-R2 selective antagonist astressin 2B (Fig. 3). Thus, our data suggests that CP-154526 induced cell death is working through the intrinsic apoptotic pathway and is associated with p53 upregulation.

### CP-154526 treatment causes an increase in intracellular Ca^2+^ levels which lead to chondrocyte cell death

**Figure 4:**
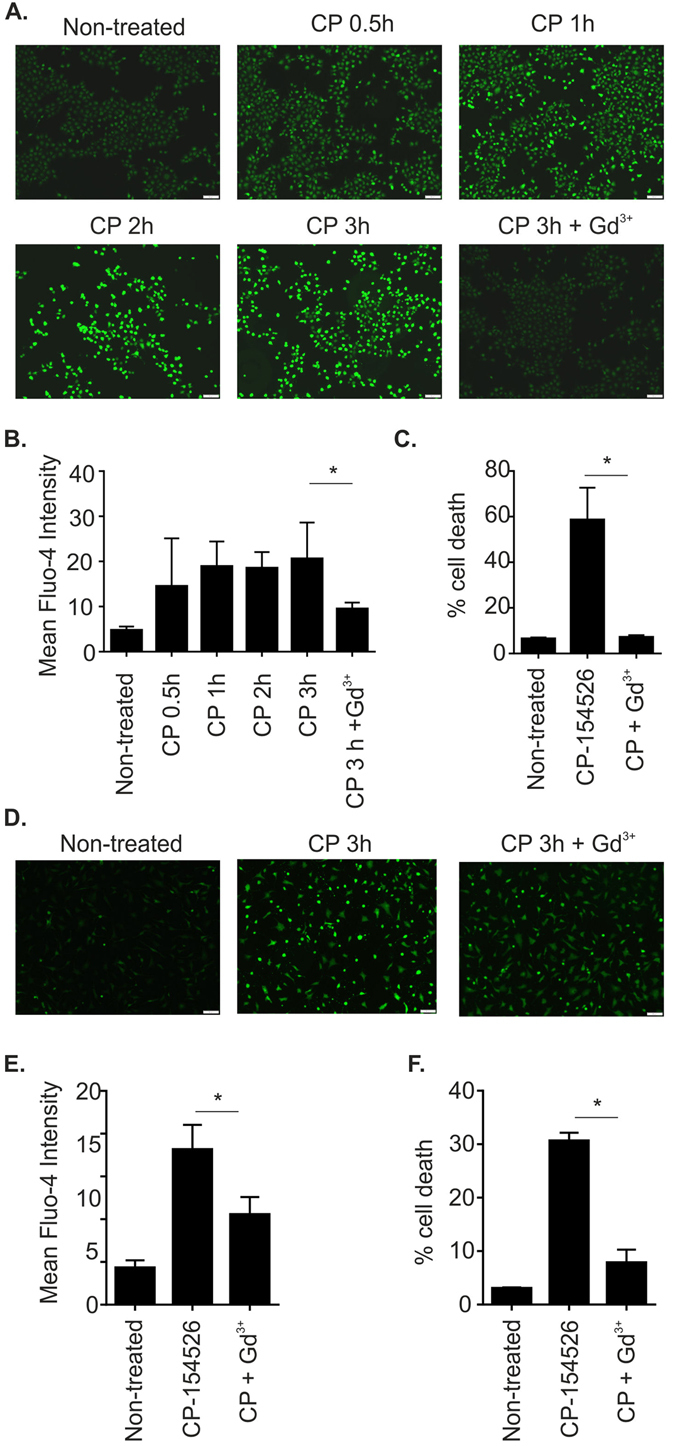
Antagonist induced calcium influx is inhibited by the non-selective cation channel blocker gadolinium. (**A**) C-20/A4 cells were treated at indicated time points CP-154526 (50 μM) alone or in the presence of Gd^3+^ (100 µM). Intracellular Ca^2+^ levels were visualised by fluorescent microscopy and Fluo-4 AM staining. Representative images of 3 independent experiments are shown. (**B**) Fluo-4 signal quantification using ImageJ software and graphical representation of Fluo-4 staining intensity for C-20/A4 cells treated with CP-154526 (50 μM) alone or in the presence of Gd^3+^ (100 µM). Mean fluorescent intensities are reported, n = 3, error bars = SD, *p < 0.05. (**C**) The ability of Gd^3+^ treatment to protect C-20/A4 cells against CP-154526 induced cell death was determined by Annexin V/PI staining and flow cytometry, n = 3, error bars = SD, *p < 0.05. (**D**) AC cells were treated with CP-154526 (50 μM) alone or in the presence of Gd^3+^ (100 µM). Intracellular Ca^2+^ levels were visualised by fluorescent microscopy and Fluo-4 AM staining. Representative images of 3 independent experiments are shown. (**E**) Fluo-4 signal was quantified using ImageJ software and graphical representation of Fluo-4 staining intensity for AC cells treated with CP-154526 alone or in the presence of Gd^3+^ (100 µM). Mean fluorescent intensities are reported, n = 3, error bars = SD, *p < 0.05. (**F**) The ability of Gd^3+^ treatment to protect primary AC cells against CP-154526 induced cell death was determined by Annexin V/PI staining and flow cytometry, n = 3, error bars = SD, *p < 0.05.

Using fluorescence microscopy we found a large increase in intracellular Ca^2+^ levels produced by the CRF-R1 antagonist from 0.5 hours to a maximum at 3 hours of incubation in C-20/A4 cells. This high level of Ca^2+^ influx was prevented with a co-treatment of the non-selective cation channel blocker Gadolinium (Gd^3+^) (Fig. 4A,B). Furthermore Gd^3+^ treatment was able to protect C-20/A4 cells from CP-154526 induced cell death (Fig. 4C). These experiments were repeated using AC where CP-154526 again caused an increase in intracellular Ca^2+^ which could be inhibited by Gd^3+^ treatment (Fig. 4D,E). Gd^3+^ was also able to protect AC against CP-154526-induced cell death.

### Ucn1 exerts its chondroprotective effect by maintaining Piezo1 in a closed conformation

**Figure 5:**
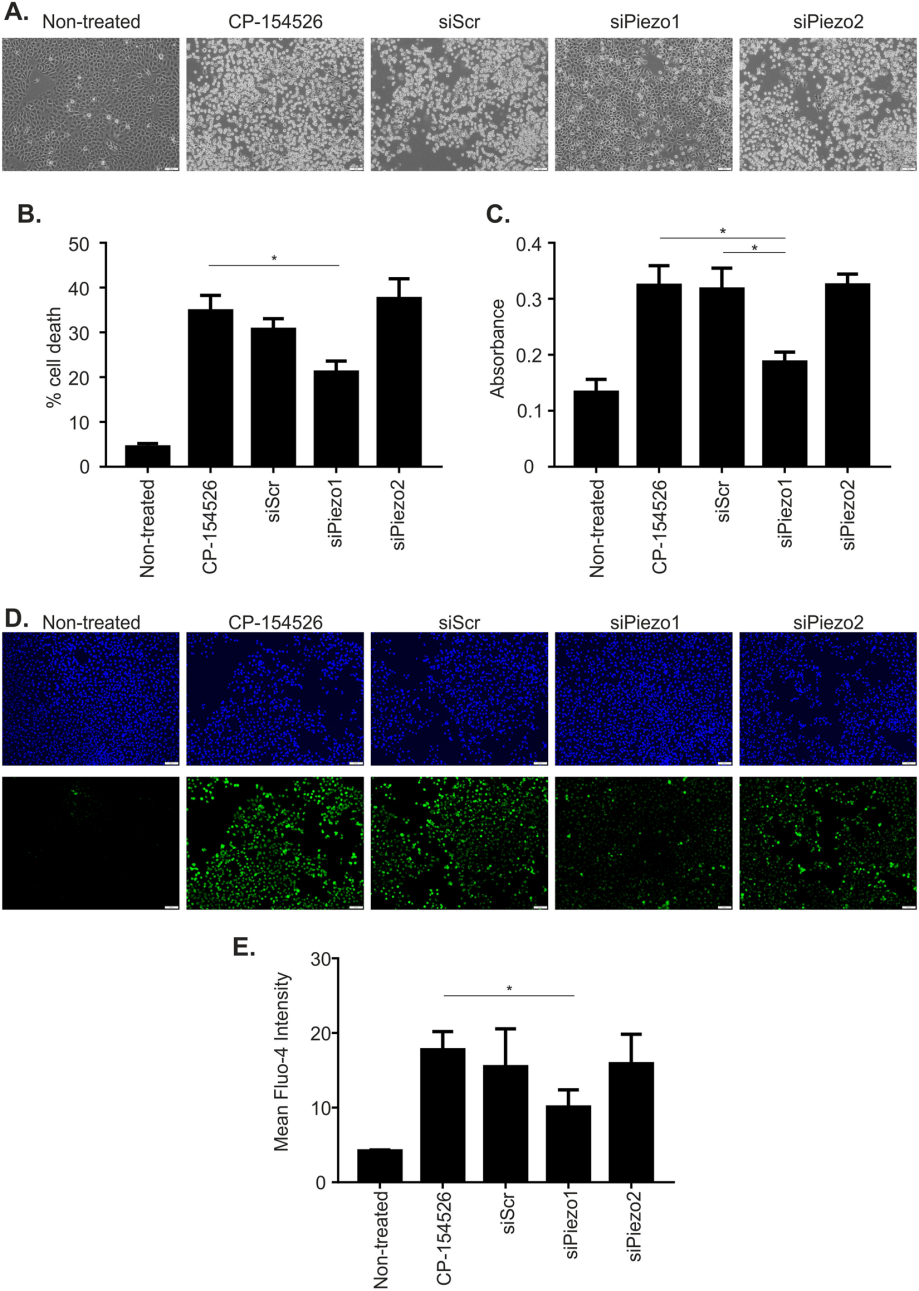
Inhibition of mechanosensitive Piezo1 can protect C-20/A4 cells from CP-154526 induced apoptosis and calcium influx (**A**). C-20/A4 cells were transfected with siRNA (20 nM) against Piezo1, Piezo2 and a Scrambled control in the presence of HiPerFect, and cultured for 72 h before treatment with CP-154526 (50 µM). Phase contrast images were collected at 5 h post-CP treatment to record changes to cell morphology. Images of the centre of each well are presented. Representative images of 3 independent experiments are shown. (**B**) Cell death in transfected and control cells was assessed 5 h post CP treatment by AnnexinV/PI staining and flow cytometry, n = 5, error bars = SEM, *p < 0.05. (**C**) Cell death measured by LDH (absorbance at 490 nm) release into the media. n = 5, error bars = SEM, *p < 0.05. (**D**) Intracellular Ca^2+^ levels were visualised in control and transfected C-20/A4 cells by fluorescent microscopy following staining with Fluo-4 AM and Hoescht-33342. Cells were treated for 3 h with CP-154526 (50 µM). Representative images of 3 independent experiments are shown. (**E**) Fluo-4 AM intensity was quantified on ImageJ software to assess calcium influx in all cells. Mean fluorescent intensities are reported, n = 3, error bars = SEM, *p < 0.05.

Using a panel of siRNA constructs we demonstrated that only silencing of the Piezo channels caused protection from cell death induced by CP-154526 as measured both by Annexin V/PI staining and LDH release. This effect was more pronounced for Piezo1 than Piezo2 (Supplementary Figure S1). From this initial study, we gained an insight into the nature of the channel involved in antagonist induced cell death. Next we repeated these initial experiments using siRNA to Piezo1 and Piezo2 and found that only when Piezo1 was lost did we see significant protection from CP-154526-induced cell death (Fig. 5A–C). We subsequently determined by silencing Piezo1 and Piezo2, that Piezo1 is responsible for CP-154526-induced Ca^2+^ influx and overload (Fig. 5D,E).

### Ucn1 binding to CRF-R1 promotes cell survival via cAMP activation and inhibition of PLA_2_ activity

**Figure 6:**
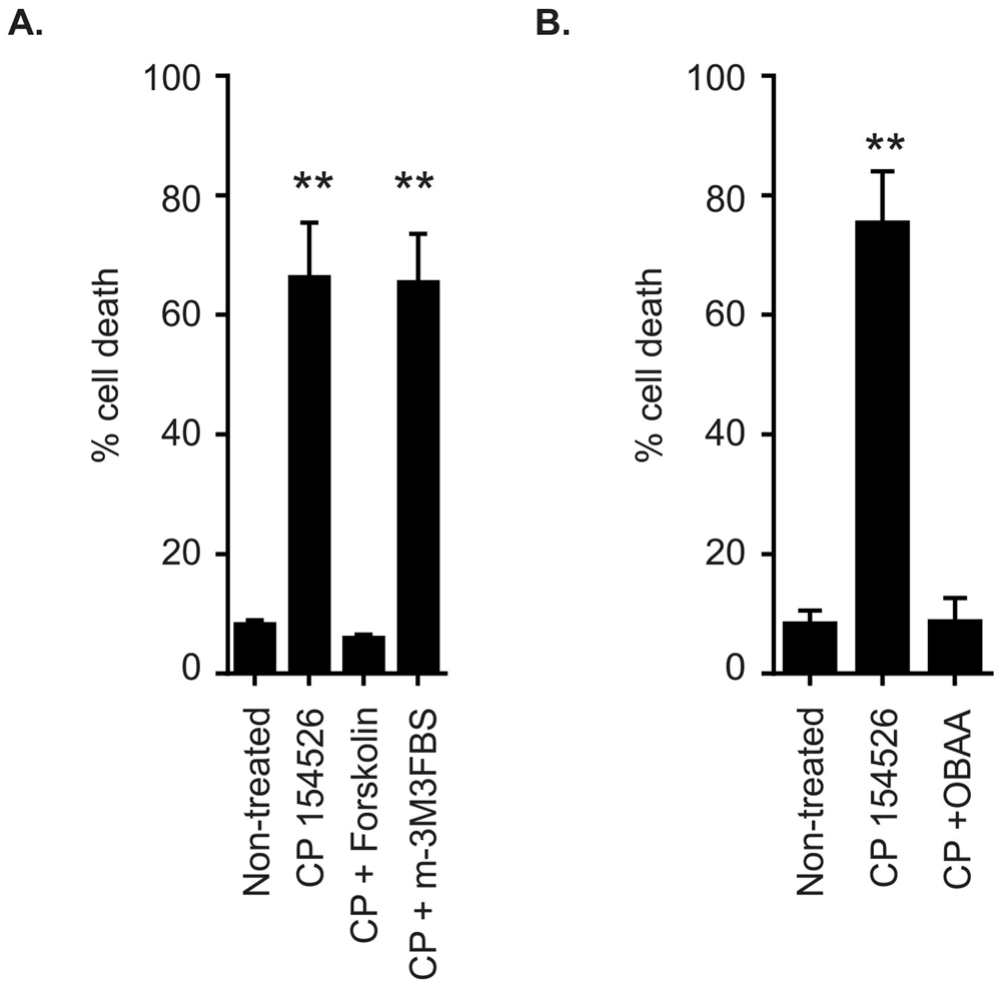
Identification of downstream signalling associated with CRF-R1. (**A**) The effect of the adenylate cyclase activator forskolin (0.1 μM) and the PLC activator m3M3FBS (0.1 μM) on CP-154526 (50 μM) induced C-20/A4 cell death as determined by Annexin V/PI staining and flow cytometry. n = 3, error bars = SD **p < 0.005 compared with control. (**B**) The effect of the PLA_2_ inhibitor OBAA (0.1 μM) on CP-154526 (50 μM) induced C-20/A4 cell death as determined by Annexin V/PI staining and flow cytometry. n = 3, error bars = SD **p < 0.005 compared with control (untreated).

Due to the promiscuity of CRF receptor coupling to different G proteins, we determined whether the coupling in our system was to the Gαs or Gαq subunits. We, therefore, investigated the possibility that C-20/A4 cells could be rescued from CP 154526 induced cell death by activating these pathways. The adenylate cyclase activator forskolin and the phospholipase C (PLC) activator m-3M3FBS were used to activate cAMP and PLC signalling, respectively, in the presence of CP-154526, thereby bypassing receptor activation by Ucn1. When this was performed we found that forskolin but not m-3M3FBS was able to rescue cells from CP-154526 induced cell death. Thus, in C-20/A4 cells Ucn1 binds to CRF-R1 and subsequently signals through adenylate cyclase activation and the generation of cAMP is required to maintain cell survival (Fig. 6A).

The phospholipase A_2_ (PLA_2_) enzyme and its metabolites can modulate numerous ion channel species, we therefore investigated the role of PLA_2_ in antagonist-induced cell death. When cells were treated with CP-154526 and the PLA_2_ inhibitor, OBAA, we found that inhibition of PLA_2_ activity rescued C-20/A4 cells from CP-154526 induced cell death. This suggested that lipid modulator production by PLA_2_ may have a role in antagonist induced cell death by causing a non-selective cation channel to become opened in the absence of Ucn1 (Fig. 6B).

## Discussion

This is the first study to demonstrate the presence of the Ucn1 system in AC (Fig. 1) and to demonstrate that endogenously released Ucn1 is essential for their maintenance and survival in the absence of externally applied noxious stimuli. Our data implies an essential pro-survival paracrine/autocrine role for Ucn1 in human chondrocytes (Fig. 2). Previously, we reported that abrogating the action of endogenously released Ucn1 with an Ucn1 depleting antibody or with the pan CRF receptor antagonist, α-helical-CRF, led to significant cell death in C-20/A4 cells^10^. Here, we used the selective CRF-R1 and R2 antagonists CP-154526, and astressin 2B, respectively, to determine the receptor subtype responsible for this pro-survival effect of Ucn1 and found that CP-154526 caused a dose dependant increase in antagonist-induced cell death in both C-20/A4 cells and AC. However, astressin 2B had no effect, demonstrating that CRF-R1 is the receptor responsible for transducing the pro-survival effect of Ucn1. Furthermore, when CP-154526 and astressin 2B were given together, no further increase in cell death was detected; possibly suggesting that CRF-R1 is the sole receptor responsible for this effect (Fig. 2). The role of CRF-R2 in these cells is as yet unknown.

Blockade of CRF-R1 caused apoptotic cell death as determined by Annexin V/PI staining and by using Western immunoblotting along with antagonist-induced cell death, we were able to demonstrate that CP-154526 but not astressin 2B caused an upregulation of p53 protein levels. As a transcription factor, p53 trans-activates a series of target genes known to inhibit cell proliferation and induce apoptosis in response to various stressors^41^. p53 expression and apoptosis also correlate with cartilage destruction in OA^5, 42^. Furthermore, in our system CP-154526 also caused cleavage of both the initiator caspase 9 and the effector caspase 3, but not caspase 8, suggesting that in the absence of Ucn1, this antagonist induced cell death is working through the intrinsic or mitochondrial apoptotic pathway (Fig. 3). Significantly, Ucn1 has also been demonstrated to prevent mitochondrial damage caused by pro-apoptotic stimuli in other cells^43^.

Ucn1 is intimately linked to the regulation of ion channels in many cell types and under different physiological conditions^24–28^. Therefore, in this study, we hypothesised that Ucn1 signalling may also exert its protective effect via blockade of a cation channel and prevent excessive Ca^2+^ overload. In chondrocytes several studies have demonstrated that excessive Ca^2+^ overload leads to cell death^44, 45^. To investigate this hypothesis we used the Ca^2+^ indicator dye Fluo-4 AM and demonstrated an increase in intracellular Ca^2+^ in the presence of CP-154526 in both our chondrocyte cell line and AC respectively (Fig. 4A–E). Furthermore, this increase in intracellular Ca^2+^ was associated with an increase in cell death and the ability of Gd^3+^ to both prevent this increase in Ca^2+^ and cell death suggest a role for a non-selective cation channel in CP-154526 induced cell death (Fig. 4C,F). The detection of an increase in intracellular Ca^2+^ influx implicates a loss of calcium homeostasis in CP-154526 induced cell death. In agreement with this is the finding that the Ucn1 homologue CRF has been shown to protect neurons against insults relevant to Alzheimer’s disease by stabilising neuronal Ca^2+^ homeostasis. In addition, Ucn1 had also been demonstrated to protect neurons against excitotoxic insults^46–48^.

We extended our investigation further in C-20/A4 cells to determine the identity of this Ucn1 regulated, Gd^3+^ sensitive cell death and Ca^2+^ influx. We used a panel of siRNAs directed towards 16 candidate ion channels commonly found in and exerting known functions on chondrocytes. Following transfection, the effect of CP-154526 on C-20/A4 cell death was assessed by both AnnexinV/PI staining and LDH release (Supplementary Figure S1). We found that only those cells with attenuated levels of Piezo1 and Piezo2 protein were protected from CP-154526 induced cell death, suggesting a role for these ion channels in cell death and Ca^2+^ overload, as both channels are known to gate calcium under conditions of compressive load^34^. We performed repeat experiments using siRNA for Piezo1 and Piezo2 and found that only Piezo1 knock down gave significant protection from both CP-154526 induced cell death and CP-154526 induced Ca^2+^ overload (Fig. 5). This data strongly implicates Piezo1 as the channel responsible for the cell death seen when Ucn1 is prevented from activating CRF-R1. As referred to in our introduction, C-20/A4 cells are acknowledged to be a good model for primary chondrocytes^39^. In this study, the results obtained from C-20/A4 cells were consistently confirmed in primary chondrocytes. Therefore, although our siRNA experiments were performed on C-20/A4 cells, we feel confident that Piezo1 is the ion channel involved in Ucn1 chondroprotection. In agreement with our study, Piezo1 was found to induce apoptosis of human chondrocytes following a compressive load which involved the MAPK/ERK1/2 signalling pathway^49^. Furthermore, Piezo1 can sense both mechanical crowding and stretch, suggesting it may act as a homeostatic sensor to control cell number, triggering extrusion and apoptosis in crowded regions and cell division in sparse regions^50^.

We went on to determine the signalling pathway involved in this Piezo1 dependant cell death. CRF receptors have been reported to be highly promiscuous with regard to their second messenger signalling pathways and can bind to multiple G-protein isoforms depending on the cell type and circumstance. Upon agonist activation, CRF receptors predominantly activate either Gαs or Gαq resulting in the generation of cAMP via adenylate cyclase, or IP_3_ and diacylglycerol via PLC^51–53^. We found that the activation of adenylate cyclase by forskolin but not the activation of PLC with m-3M3FBS, was able to rescue chondrocytes from antagonist-induced cell death, demonstrating that CRF-R1, when bound to Ucn1 in chondrocytes, is coupled to Gαs and is responsible for the generation of cAMP, and therefore the regulation of protein kinase A (PKA) activity (Fig. 6A). Recently, Piezo2 current amplitude was demonstrated to be increased, and inactivation was slowed, by bradykinin 2 receptor (BDKRB2) activation in heterologous expression systems. This G-protein coupled receptor produced this effect by activating both PKA and PKC, whereas the effect was abolished by PKA and PKC inhibitors^54^. Although the effect of BDKRB2 agonism was to activate a Piezo channel rather than to produce an inhibitory effect, (as seen with Ucn1), clearly there is a role for G-protein coupled receptor activation in the regulation of Piezo channels. Indeed inhibition of Piezo1 and Piezo2 was found to be caused by depletion of phosphatidylinositol 4,5-bisphosphate and its precursor PI(4)P from the plasma membrane by Ca^2+^ - induced phospholipase Cδ activation^55^. These findings imply a role for membrane bound lipid factors in channel regulation. Our results clearly demonstrate, however, that adenylate cyclase, rather than PLC, is involved in Piezo1 regulation by Ucn1. Several known endogenous modulators of non-selective cation channels include lipid based ligands derived from enzymatic catabolism of arachidonic acid (AA) by cytochrome P450 epoxygenase^56–58^. AA itself is derived from the degradation of membrane phospholipids usually by PLC. An alternative route for the derivation of lipid modulators during antagonist induced cell death is the generation of AA by PLA_2_^59^. Indeed, using the PLA_2_ inhibitor OBAA, we were able to demonstrate that antagonist induced cell death involved activation of PLA_2_, as its inhibition was protective when CP-154526 was applied to chondrocytes (Fig. 6B). This implies that activation of PLA_2_ is required for CP-154526 induced cell death and that when Ucn1 is present, PLA_2_ is inactive, thereby preventing the generation of endogenous lipid based channel openers. Significantly, Ucn1 has been demonstrated to both down-regulate calcium-independent PLA_2_ (iPLA_2_)^60^ and regulate its activity^61^ in other systems during cell stress. Of note, an iPLA_2_ type enzyme producing AA and lysophospholipids from the plasma membrane, has been demonstrated to modulate a store operated channel activity by regulating iPLA_2_ kinetics through cAMP/PKA dependant rather than PLC dependant mechanisms^62^. Furthermore, multiple isoforms of PLA_2_ have been revealed to participate in inflammatory processes in OA^63^, further implicating this pathway in the progression of OA.

**Figure 7:**
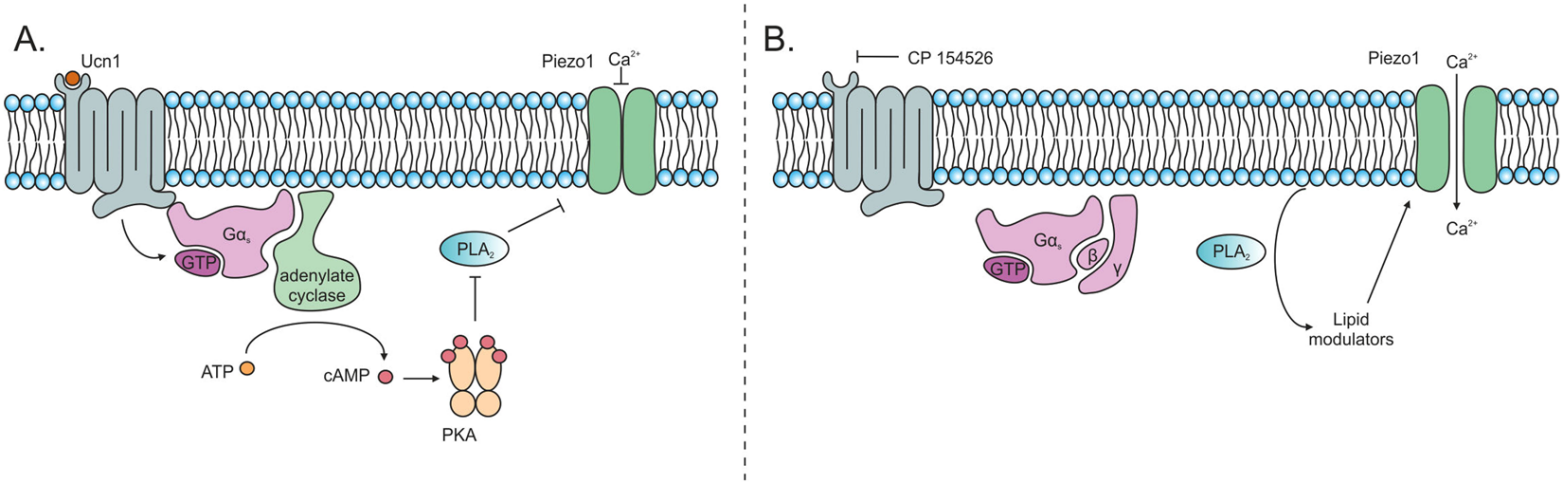
Schematic diagram depicting the mechanisms by which Ucn1 promotes chondrocyte cell survival. (**A**) During chondrocyte cell survival, Ucn1 is bound to CRF-R1 in an autocrine/paracrine fashion. This causes recruitment of Gαs which activates adenylate cyclase, producing elevated levels of cAMP. cAMP then activates PKA which causes the inactivation of PLA_2_ reducing the breakdown of membrane phospholipids, thereby reducing the production of ion channel openers. This results in Piezo1 remaining in a closed conformation, preventing Ca^2+^ overload. (**B**) However, if the agonist effect of Ucn1 for CRF-R1 is removed, in this case by the selective antagonist CP-154526, Gαs is not recruited by CRF-R1 and therefore adenylate cyclase is not activated resulting in a reduction in cAMP levels. This then causes the inactivation of PKA which results in the removal of its block on PLA_2_ which therefore becomes activated, increasing the generation of potential lipid openers of the Piezo1 channel. If this situation is maintained, the cell experiences a Ca^2+^ overload and ultimately cell death.

Here, we have dissected the signalling pathway from the CRF receptor to the regulation of Piezo1. This involves both cAMP and PLA_2_, and demonstrates that Ucn1 is crucial for indirect gating of this channel. Importantly, removal of the CRF-R1 block, causes persistent and excessive opening of Piezo1 resulting in Ca^2+^ overload and subsequent cell death (Fig. 7). Therefore, the bioavailability and concentration of Ucn1 and factors affecting this would be of crucial importance to maintain correct calcium homeostasis in chondrocytes. This would be of immense interest in understanding and exploiting chondrocyte cell fate in health and disease. Our study is the first to demonstrate a role for Ucn1 in the regulation of a mechanosensitive channel, Piezo1. Therefore, the label ‘mechanosensitive channel’ belies its more complex regulatory nature, in that as well as being gated by mechanical force, Piezo1 in chondrocytes is also regulated by intracellular signals generated by a G-protein coupled receptor. We have previously found that Ucn1 can protect chondrocytes from both pro-apoptotic factors associated with OA and acute impact injury (Unpublished observations). Ucn1, therefore, may represent a link between cellular derived factors and injurious factors both capable of the production of OA through different pathways. Much work is still required to fully elucidate this novel chondroprotective pathway. However, once found, it will open up new approaches for the development of novel treatments that could prevent or modulate the progression of OA, rather than just addressing the symptoms of the disease.

## Materials and Methods

### Cell culture

The C-20/A4 human chondrocyte cell line was cultured in a medium consisting of Dulbecco’s modified Eagle’s medium (Invitrogen, UK), supplemented with 10% (v/v) foetal calf serum (FCS, UK)1% (v/v) and 1% (v/v) penicillin/streptomycin (Invitrogen, UK) at 37 °C, 5% CO_2_. Cells were passaged by treatment with Ca^2+^ and Mg^2+^-free phosphate-buffered saline, pre-warmed to 37 °C and used as required. Following passage, the chondrocyte cell suspension (1 × 10^6^ cells/ml) was transferred to six-well tissue culture plates and incubated for 24–48 h in the above medium to allow cells to reach ∼80% confluency. The cells were then ‘serum starved’ for 24 h by replacing the normal, 10% FCS growth medium with medium containing 1% FCS (all other components were unchanged). Cells were then treated as indicated in specific experiments.

### Primary human chondrocytes isolation and culture

These methods and experimental protocols were carried out in accordance with the approved guidelines as approved by the National Research Ethics service and the University of Manchester ethics committee number 10/H1013/27. Written informed consent was obtained from all patients. Femoral heads were collected from patients undergoing total hip or knee replacement surgery and stored in serum-free DMEM with antibiotics/antimycotics until processed (Supplementary Table S1). Femoral head cartilage was shaved off using a scalpel and incubated overnight with agitation in serum-free DMEM containing an enzymatic cocktail of 0.25% collagenase type II (Life Technologies) and 0.1% hyaluronidase in serum-free medium (Sigma) to release the AC. The next day the solution was centrifuged and the pelleted ACs resuspended and plated in DMEM supplemented with 10% foetal calf serum (FCS) at a density of approximately 5,000–6,000 per cm^2^. Cells were then incubated for 24–48 h in the above medium to allow cells to reach ∼80% confluency. The cells were then ‘serum starved’ for 24 h by replacing the normal, 10% FCS growth medium with medium containing 1% FCS (all other components unchanged). Cells were then treated as indicated in specific experiments.

### RNA isolation and RT-PCR

Total RNA was isolated using Qiagen RNeasy RNA isolation kit (Qiagen) and Total RNA was subjected to on-column RNase-free DNase digestion. RNA purity and concentration were determined spectrophotometrically at 260 and 280 nm using a NanoDrop 2000c spectrophotometer (Thermo Scientific). RNA integrity was verified by 1% agarose gel (containing GelRedTM) electrophoresis. The gel was visualised on a UV transilluminator. cDNA was synthesised from 2 µg of RNA using oligo dT primer in a 20 μl first-strand cDNA synthesis reactions containing 18 units of AMV reverse transcriptase (Promega). The reaction was terminated by incubation at 94 °C for 3 minutes and then stored at −20 °C until use. Amplification was performed with 3 μl of cDNA for GAPDH, Ucn1, CRF-R1 and CRF-R2 under conditions described in Supplementary Table S2. Amplified PCR products were analysed on agarose gels.

### Pharmacological treatment of cells

Six-well plate cultures of either the C-20/A4 cell line or AC were treated for 8 h with individual or combination treatments of: the CRF-R1 specific antagonist CP-154526 (1–50 μM; Tocris), the CRF-R2 specific antagonist astressin 2B (1–50 μM; Tocris), the non-selective cation channel blocker Gd^3+^ (100 μM; Tocris), the adenylate cyclase activator forskolin (0.1 μM; Tocris), the PLC activator m3M3FBS (0.1 μM; Tocris), or the PLA_2_ inhibitor OBAA (0.1 μM; Tocris). Cells were then assayed for cell death or photographed using an Olympus IX73 phase contrast microscope and camera.

### Transient transfection of C-20/A4 cells

A panel of siRNA’s against candidate ion channels and controls was purchased from Dharmacon (GE Dharmacon) each siRNA was a pool of 4 sequences (Supplementary Table S3). All siRNA was reconstituted to 20 µM stock concentration in RNase-free water. C-20/A4 cells were plated at a density of 250,000 cells/well of a 6 well plate in 10% FCS media as described above, and incubated at 37 °C and 5% CO_2_ for 30 minutes. Reconstituted siRNA was diluted to 20 nM working concentration in 200 μl of Opti-MEM (Gibco). 12 µl of HiPerFect (Qiagen) was added to the siRNA and Opti-MEM and incubated for 15 minutes at room temperature. Following incubation, siRNA solution was added to cells in a dropwise manner and incubated in normal culture conditions for 48 h. 12 h before collection, media was changed on all wells and replaced with 1% FCS media. siPiezo1 and siPiezo2 of the same pooled sequences was purchased from Dharmacon for further experiments. C-20/A4 cells were transfected with 20 nM siRNA against Piezo1, Piezo2 and a Scrambled control in the presence of HiPerFect, and cultured for 72 h before treatment with CP-154526 (50 µM). Phase contrast images were collected at 5 h post-CP treatment.

### Detection of cell death- flow cytometry and Lactate dehydrogenase (LDH)

Cell death was detected using the Annexin V-FITC Apoptosis Detection Kit I (BD Bioscience) as per the manufacturer’s instructions. Cells were harvested by trypsinisation, washed in PBS and resuspended in binding buffer containing 5 µl of annexin V-FITC and 5 µl PI staining solution and incubated for 15 minutes at room temperature in the dark. Cells were then analysed on a BD Accuri C6TM flow cytometer. Data was analysed using BD Accuri C6TM software.

LDH assays were conducted on media collected at 5 h post-treatment with CP-154526 (Pierce™ LDH Cytotoxicity Assay Kit, Thermo Fisher Scientific, catalogue number 88954). Media was thoroughly agitated and 50 µl added to wells of a 96-well plate in duplicate. 50 µl reaction buffer was added to each sample, and mixed by pipetting 5 times. The plate was then incubated in the dark at room temperature for 30 minutes. Reaction was stopped with 50 µl stop solution provided with the kit. Absorbance was recorded at both 490 nm and 680 nm on SpectraMax M5 plate reader (Molecular Devices, CA, USA). Positive controls provided with the kit and negative controls in the form of culture media alone were conducted with every experiment. Readings were adjusted for background fluorescence by subtracting the value recorded at 680 nm from that recorded at 490 nm for each sample. Final values presented are an average of the duplicated readings with background absorbance reading subtracted.

### Immunoblotting

Cells were harvested using trypsin digestion and lysed using RIPA buffer with protease inhibitor cocktail (Sigma). Total protein was quantified using BCA protein assay kit (Pierce) and equal quantities of denatured protein were subjected to electrophoresis on SDS-polyacrylamide gels, blotted onto Immobilon-FC transfer membrane (Sigma) and probed with the following specific primary antibodies; anti-p53 (Cell Signalling Technology, 9282), anti-cleaved caspase 3 (EMD Millipore, AB3623), anti-cleaved caspase 9 (EMD Millipore, AB3629), and anti-GAPDH (Abcam, ab8245). Blots were washed and probed with the following appropriate secondary antibodies; goat anti-rabbit IgG IRDye 800CW (IRDye antibodies, 926–32211), goat anti-mouse IgG IRDye 800CW (IRDye Antibodies, 926–32210) or goat anti-rabbit IgG IRDye 680LT (IRDye Antibodies, 926–68021). The signal was visualised using a LICOR Odyssey imaging system.

### Analysis of intracellular Ca^2+^ levels

Intracellular Ca^2+^ levels were visualised using fluorescent microscopy and Fluo-4 AM permeant dye (Thermo Fisher Scientific). After incubation with Gd^3+^ (100 μM) and CP-154526 (50 μM), cells were incubated within media containing 1 µM Fluo-4 AM and 5 μg/ml Hoechst 33342 for 30 minutes at 37 °C before being washed twice with HEPES buffered saline solution and imaged using an Olympus IX73 fluorescent microscope.

### Statistical analysis

Data values from the figures are expressed as mean ± standard deviation of the mean. All data were subjected to a one way ANOVA followed by a Tukey HSD test where appropriate. Probabilities of p < 0.05 were considered statistically significant. Statistical analysis was performed using GraphPad Prism.

## Electronic supplementary material

Supplementary data
