# Supplementary material for: Chondroprotection by urocortin involves blockade of the mechanosensitive ion channel Piezo1
Source: Sci Rep. 2017 Jul 11;7:5147. doi: 10.1038/s41598-017-04367-4 (PMC5505992; doi:10.1038/s41598-017-04367-4)
Supplement: Supplementary file 1 — Supplementary data [file 41598_2017_4367_MOESM1_ESM.pdf]

Chondroprotection by urocortin involves blockade of the mechanosensitive ion channel Piezo1.

K.M. Lawrence*[1], R.C. Jones[1], T.R. Jackson[1], R.L. Baylie[2], B. Abbott[1], B. Bruhn-Olszewska[1], T.N. Board[3], I.C Locke[4], S.M. Richardson[5], P.A Townsend[1]

Supplementary information

Gender	Age	Joint
Female	47	Hip
Female	56	Hip
Male	68	Hip
Female	76	Knee
Female	55	Knee
Female	64	Hip
Male	26	Hip
Female	55	Knee
Female	50	Hip
Male	29	Hip
Male	69	Hip

Table S1: Patient demographics.

Target Gene	Sense Primer Sequence 5’-3’	Anti-sense Primer Sequence 5’-3’	Length of fragment (bp)	PCR protocol	Cycles
**UCN1**	CAGGCGAGCGGCCGCG	CTTGCCCACCGAGTCGAAT	146	95°C/57°C/60°C	33
**CRF-R1**	ACAAACAATGGCTACCGGGA	GGACCACGAACCAGGTGGCG	280	95°C/58°C/60°C	45
**CRF-R2**	AGCCCATTTTGGATGACAAG	AGGTGGTGATGAGGAGGCC	180	95°C/58°C/60°C	45

Table S2: PCR primer sequences and conditions used to amplify the Ucn1 system components in AC.

A.

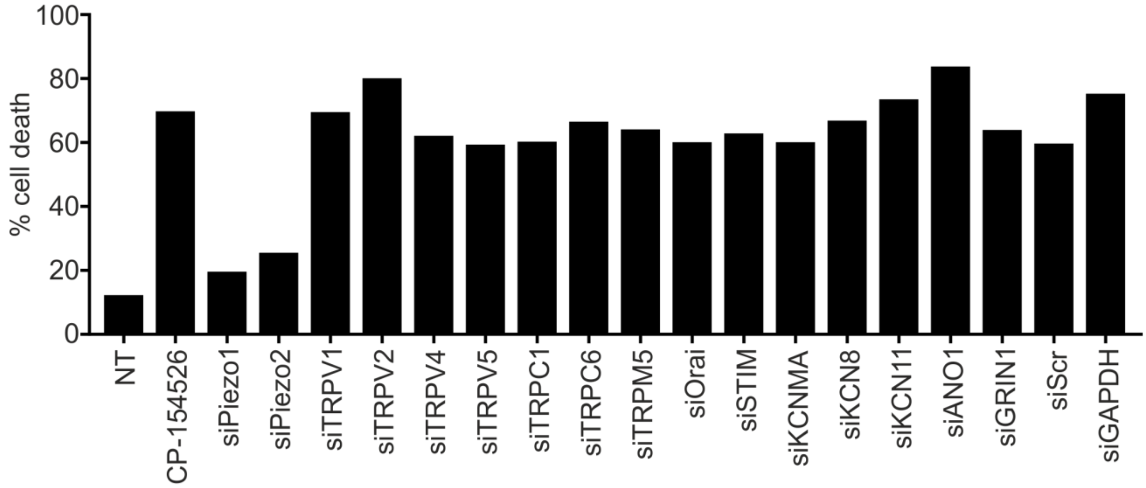

B.

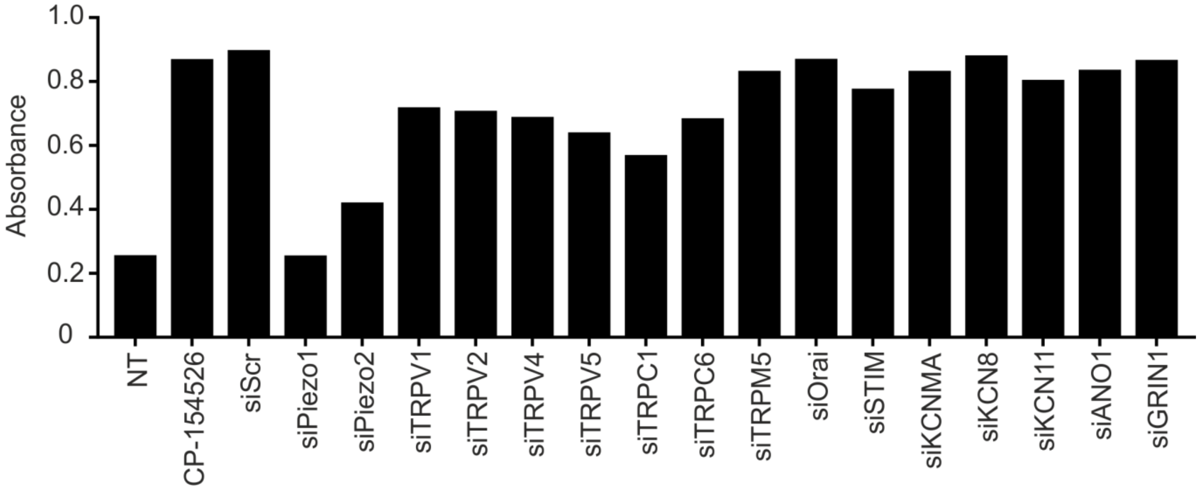

Figure S1: A bespoke panel of siRNA selected against 16 candidate ion channels derived from an extensive literature search was used to screen potential candidates for the ion channel modulated by Ucn1 in this system. C-20/A4 cells were transfected in the presence of HiPerFect and cultured for 72h before addition of CP-154526 (50µM). Cells and media were collected separately at 5h. A). Transfected C-20/A4 cells were stained with AnnexinV/PI and levels of cell death assessed by flow cytometry. Representative data for 2 independent experiments is shown. B.) Media from cells following treatment with CP-154526 was collected and LDH levels assessed at absorbance of 490nm. Mean absorbance minus background is reported, representative data for 2 independent experiments is shown.

siRNA	Sequence	siRNA	Sequence
**PIEZO1**	AAAGACAGAUGGAGCGUAU	**ORAI1**	GCUCACUGGUUAGCCAUAA
	GCAAGACCGUGCUGGGCAA		GGCCUGAUCUUUAUCGUCU
	UCGCGGUGGUCGUCAAGUA		GCACCUGUUUGCGCUCAUG
	GCAAGUUCGUGCGCGGAUU		CAGCAUUGAGUGUGUACAU
**PIEZO2**	GGAAUUCACUUCUGGAUGU	**STIM1**	CAUCAGAAGUAUACAAUUG
	GCUAUGGUAUUAUGGGAUU		AGAAGGAGCUAGAAUCUCA
	UUACGUCAACCUCUUCUUA		AGGUGGAGGUGCAAUAUUA
	CACCGGUGCUAUGCAAUUU		GGUGGUGUCUAUCGUUAUU
**TRPV1**	GGAGACUAUUUCCGAGUUA	**KCNMA1**	GAAGGAGUCUCAAAUGAAA
	UGACGAGCAUGUACAAUGA		GAACUACCAUCCGAAGAUA
	CAUCUAUGCCGUCAUGAUA		GCAAAGCUCUGAAAACAUA
	CGAGGAAGUUUAUCUGCGA		GAUCCAAGAAGGUACUUUA
**TRPV2**	GGUAAGACGUGCCUGAUGA	**KCNJ8**	GCACGAACCUCCUACAUUG
	GAAAUGGGAUCUGCUCAUC		GAGAAACCUUCCAUCCUUA
	GGCUGAACCUGCUUUACUA		GAACAUCCGUGAGCAAGGA
	GCGAGACCGUCAACAGUGU		GGUCAUAGUUAUUCUGGAA
**TRPV4**	GCACACCGCCGUACCCUUA	**KCNJ11**	GCAACGUGGCCCACAAGAA
	GACCAAAUCUGCGCAUGAA		CGCAAGAGCAUGAUCAUCA
	CAACCGGCCUAUCCUCUUU		ACAUGCAGGUGGUACGCAA
	GAACCCGUGUGCCAACAUG		GACGUUACUCUGUGGACUA
**TRPV5**	GAGAGGAGGUCUACCAUUU	**ANO1**	GCAGAGAGGCCGAGUUUCU
	GAACCACAAUGAUCAGAAU		GCACGAUUGUCUAUGAGAU
	GUAUUUCACUCGAGGAUUC		UUACGUGGCGUUCUUCAAA
	UAGCAGCCCUCUAUGACAA		GUACGCGGCUGCAUACCCA
**TRPC1**	GAACAUAAAUUGCGUAGAU	**GRIN1**	GCCGGGAUCUUCCUGAUUU
	GGACUACGGUUGUCAGAAA		AAGCAGAGCUCCGUGGAUA
	GAGAAGAACUGCAGUCCUU		GGAGCACGCUGGACUCGUU
	UCAGGUGACUUGAACAUAA		AGACGUGGGUUCGGUAUCA
**TRPC6**	GCACAAAACUCCUUCCUAA	**ON-TARGETplus GAPDH Control**	GUGUGAACCACGAGAAAUA
	GGACACGGUUCUCCCAUGA		GGAGAAACCUGCCAAGUAU
	GCAGAUAUCACUUGGAAGA		UCAAGAAGGUGGUGAAGCA
	UGAACGGCCUCAUGAUUAU		UGGUGAAGCAGGCAUCUGA
**TRPM5**	GGGCAACGCAGACAUGUUC	**ON-TARGETplus Scrambled Control**	UGGUUUACAUGUCGACUAA
	GUACUUCGCCUUCCUCUUC		UGGUUUACAUGUUGUGUGA
	GAAGAAGUUCACACUGUAU		UGGUUUACAUGUUUUCUGA
	GAGCAGCCUUUCGCCAUGA		UGGUUUACAUGUUUUCCUA

Table S3: siRNA sequences used in the transfection experiments for the 16 different ion channel species as indicated.
